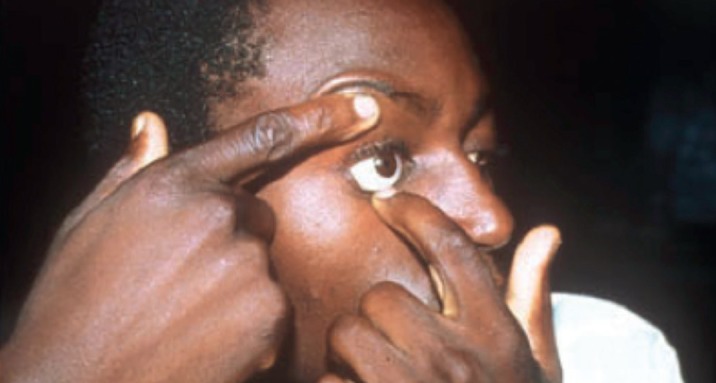# Management of an eye prosthesis or conformer

**Published:** 2013

**Authors:** Sue Stevens

**Affiliations:** Former Nurse Advisor, *Community Eye Health Journal*, International Centre for Eye Health, London, UK.

**Figure:**
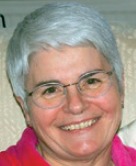
Sue Stevens

**You will need**

- Small pot of saline or cooled boiled water
- Mirror
- Cotton buds
- Gauze swabs
- Prosthesis (artificial eye) or conformer (shell)

**Before you start**

- Provide privacy so that the patient can practice this procedure without others looking on.
- Encourage the patient and reassure them that the procedure, although perhaps difficult at first, will become easier for him/her to manage alone.
- Patients may wish to observe, by using a mirror, someone performing this procedure on them before attempting removal and insertion by themselves.
- Encourage patients to look at and feel the empty socket. They may be fearful of doing so, and this is often the main challenge in building confidence for self-care.

**Inserting the prosthesis or conformer**

It will help if the patient looks downwards.

**Figure 1:**
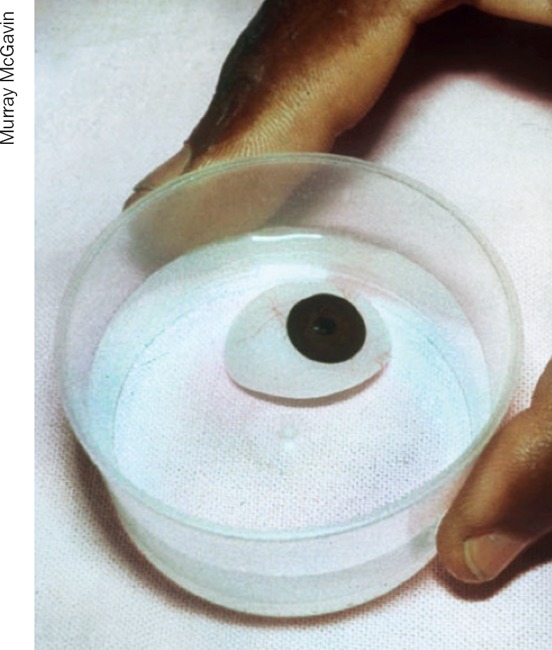


**Figure 2:**
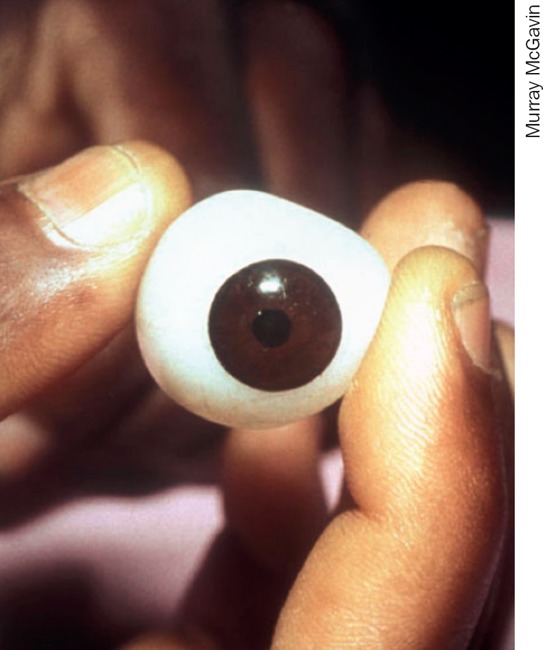


**Figure 3:**
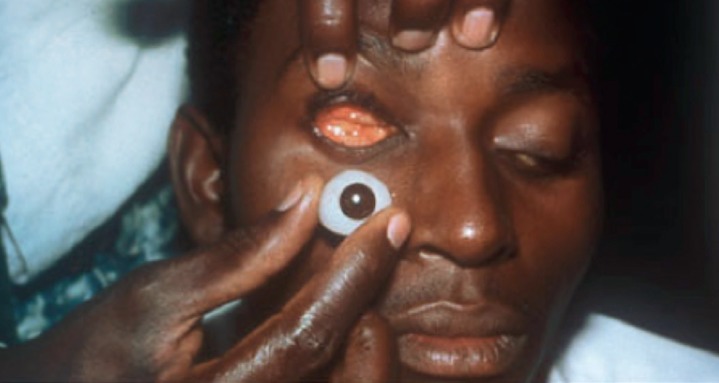


**Figure 4:**
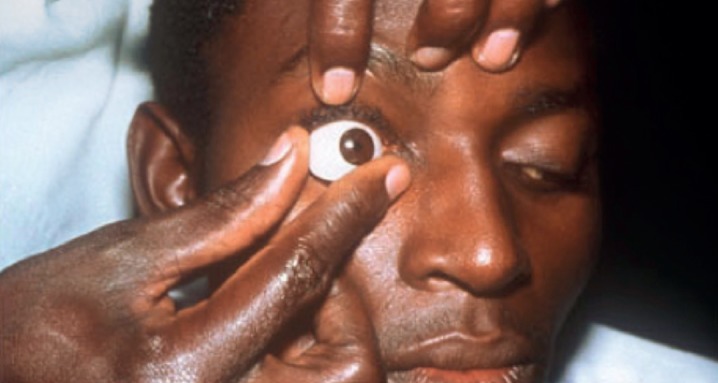


**Figure 5:**
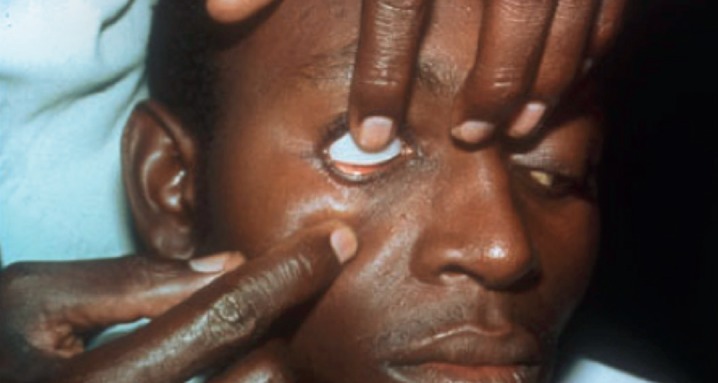


1. Clean the eyelids using cotton buds or gauze moistened in saline.
2. Take the prosthesis or conformer and moisten in the saline (Figure 1).
3. Hold the prosthesis or conformer between the thumb and forefinger with the indentation uppermost and the convex surface outermost (Figure 2).
4. Using the other hand, gently lift the upper eyelid with a fingertip (Figure 3).
5. Insert the upper part of the prosthesis or conformer under the eyelid in an upwards, backwards and inwards movement (Figure 4).
6. Remove the hand from the upper eyelid (but still support the prosthesis or conformer) and, with the free hand, gently pull down the lower eyelid. The lower part of the prosthesis or conformer should then slip easily into place inside the lower eyelid (Figure 5).
7. Check that normal eyelid closure is possible and, importantly, comfortable for the patient.

**To remove the prosthesis or conformer**

It will help if the patient looks **upwards**.

- Clean the eyelids using cotton buds or gauze moistened in saline.
- Using an index finger gently pull down the lower eyelid in order to see the edges of the prosthesis or conformer.
- Gently push the eyelid under the prosthesis or conformer and, with the other hand, exert some fingertip pressure on the upper eyelid. The prosthesis or conformer should slip out easily into the cupped hand (Figure 6). (The picture shows the patient doing this himself.)
- Place the prosthesis or conformer in the saline. It should be cleaned thoroughly before re-insertion.

**Figure 6:**